# Cortical thickness and cognitive performance in asymptomatic unilateral carotid artery stenosis

**DOI:** 10.1186/s12872-019-1127-y

**Published:** 2019-06-25

**Authors:** Alina Nickel, Simon Kessner, Andreas Niebuhr, Julian Schröder, Caroline Malherbe, Felix Fischer, Marlene Heinze, Bastian Cheng, Jens Fiehler, Hans Pinnschmidt, Axel Larena-Avellaneda, Christian Gerloff, Götz Thomalla

**Affiliations:** 1 Department of Neurology, University Medical Center Hamburg-Eppendorf, Hamburg, Germany; 2 Center for Experimental Medicine, Institute of Computational Neuroscience, University Medical Center Hamburg-Eppendorf, Hamburg, Germany; 3 Department of Neuroradiological diagnostics and intervention, University Medical Center Hamburg-Eppendorf, Hamburg, Germany; 4 Institute of Medical Biometry and Epidemiology, University Medical Center Hamburg-Eppendorf, Hamburg, Germany; 5 Department of Vascular Medicine, University Heart Center Hamburg GmbH (UHZ), Hamburg, Germany; 6 Klinik und Poliklinik für Neurologie Kopf- und Neurozentrum, Universitätsklinikum Hamburg-Eppendorf, Martinistr. 52, 20246 Hamburg, Germany

**Keywords:** Cortical thickness, Carotid artery stenosis, MRI, Cognitive

## Abstract

**Background:**

We investigated changes of cortical thickness and its association with cognitive performance in patients with high-grade carotid artery stenosis without ischemic brain lesions.

**Methods:**

We studied 25 patients with unilateral carotid artery stenosis ≥50% and 25 age-matched controls. All subjects underwent T1-weighted MRI, and cortical thickness was measured in 33 regions of interest in each hemisphere, as well as in brain regions belonging to the vascular territory of the middle cerebral artery (MCA). General linear mixed models were fitted to the dependent variable cortical thickness. Cognitive assessment comprised the Stroop Test and Trail Making Test B.

**Results:**

In the linear mixed model, presence of carotid stenosis had no effect on cortical thickness. There was a significant interaction of stenosis and region with a trend towards lower cortical thickness in the MCA region on the side of carotid stenosis. Patients with carotid stenosis performed significantly worse on the Stroop test than controls, but there was no correlation with cortical thickness.

**Conclusion:**

In patients with carotid stenosis without ischemic brain lesions, neither a clear pattern of reduced cortical thickness nor an association of cortical thickness with cognitive function was observed. Our data do not support the hypothesized association of cortical thinning and cognitive impairment in carotid stenosis.

**Electronic supplementary material:**

The online version of this article (10.1186/s12872-019-1127-y) contains supplementary material, which is available to authorized users.

## Background

Carotid stenosis is a known risk factor for stroke [1]. In population based studies, asymptomatic carotid stenosis can be found in up to 5.7%, with higher prevalence in men and with higher age [2, 3]. While carotid stenosis is usually termed “asymptomatic” in the absence of stroke related to the stenosis, there is continuous debate about detrimental effects of carotid stenosis on brain structure and cognition beyond the occurrence of cerebrovascular events.

Non-invasive measurement of cortical thickness by structural MRI has come into focus as a biomarker of brain pathology as it was linked with performance in cognitive tests [4]. It is considered to reflect the structural integrity of cortical gray matter, and can be influenced by age, sex, subcortical lesions, specific training or diseases of the brain [5, 6]. Vascular risk factors were also found to be associated with cortical thinning [7].

Hemodynamic compromise with focal brain lesions, i.e. ischemic stroke, is well known to go together with secondary cortical thinning [8–10]. The same was observed for symptomatic hemodynamic impairment, e.g. subclavian steal syndrome [11]. For asymptomatic carotid stenosis, data on cortical thickness is scarce. A pilot study suggested, that altered cerebral perfusion in carotid stenosis is associated with regional cortical thinning [12]. Another study revealed a decrease of cortical thickness specifically in the anterior cerebral artery territory [13]. Several studies report an association of carotid artery stenosis with impaired cognitive performance [14–22]. Whether structural brain changes including regional cortical thinning represent a relevant mechanism contributing to cognitive impairment in carotid stenosis remains unclear.

We aimed at evaluating cortical thickness and a hypothesized association of cortical thinning with impaired cognitive function in patients with unilateral carotid stenosis in the absence of focal ischemic brain lesions.

## Methods

### Patients and controls

Between March 2015 and October 2016, we enrolled 25 patients with findings of unilateral carotid artery stenosis without ischemic brain lesions on MRI, in whom interventional or surgical treatment of carotid stenosis was planned. Patients presented to the outpatient departments or were referred for treatment at the Department of Neurology or at the Department of Vascular Surgery. Decision to perform carotid revascularization was made by interdisciplinary consensus decision as described recently [23]. Inclusion criteria were: unilateral internal carotid artery stenosis of at least 50% assessed by ultrasound following the North American Symptomatic Carotid Endarterectomy Trial (NASCET) [24, 25]; between 60 and 90 years of age; no history of stroke; no ischemic brain lesion on MRI. As a control group, we studied 25 healthy age-matched controls. Inclusion criteria were no history of a TIA or stroke and no neurological or psychiatric disease. Exclusion criteria for both groups were a history of a severe disease, structural brain lesions and inability to undergo MRI. All participants provided written informed consent. The study was approved by the ethics committee of the Hamburg chamber of physicians.

### MRI protocol and image analysis

MRI was performed prior to treatment of the stenosis on a 3 T Skyra MRI scanner (Siemens, Erlangen, Germany). For anatomical imaging, a T1-weighted high-resolution three-dimensional magnetization-prepared, rapid acquisition gradient-echo sequence (MPRAGE) was used with the following parameters: TR = 2500 ms, TE = 2.12 ms, FOV = 240 × 192 mm, 256 axial slices, ST = 0.94 mm, IPR = 0.94 × 0.94 mm.

T1-weighted MR-images were preprocessed using the Freesurfer software package 6.0.0 with standard procedures and parameters [26–28]. After careful visual inspection of images and skull stripping and registration to MNI space coordinates [29, 30], Freesurfer segments the brain volume into white and grey matter creating white matter-gray matter and pial surface boundary. From these volumes, the cortical thickness is calculated for 35 labelled region of interests (ROIs) on the cortical surface. For our analysis, we excluded the entorhinal ROI resulting from its eccentric position making it prone to artifacts and inaccurate results. Thus, we included 33 ROI in our analysis.

In addition, we defined vascular regions of interest by brain regions in the vascular territory of the middle cerebral artery (MCA) according to previous description of arterial territories [31–33]: (MCA region), and all other brain regions (non-MCA region).

### Cognitive tests

Cognitive tests were performed on the same day as the MRI examination and comprised the following: Stroop Test and trail making test B (TMT B), being part of the extended Consortium to Establish a Registry for Alzheimer’s disease (CERAD-Plus) test battery. From the results of the TMT B [34], z scores were determined according to evaluation system CERAD-Plus Online (https://www.memoryclinic.ch/de/main-navigation/cerad-plus/), of the Memory Clinic (Universitäre Altersmedizin, Felix Platter-Spital, Basel, memoryclinic@fps-basel.ch) [42]. The CERAD-Plus online analysis program transforms the total raw score into specific values and converts them into z scores that are corrected for age, education and sex, thereby enabling comparison with performance of a standard reference sample. Z scores describe by how many standard deviations the results differ from the reference sample. By subtracting the time to accomplish the Farb-Wort- Test (FWT, corresponding to the Stroop Test) II and III, results were obtained that were modified according to the age of each patient by assessment tables from the Nürnberger Alters-Inventar [35], thereby resulting in C scores. Additionally, the Mini Mental State Examination (MMSE) was performed by all participants and the clock drawing test (CDT) and dementia detection test (DemTect). Higher score in the MMSE and DemTect are reflected by better cognitive function, whereas higher score in the CDT is associated with lower cognitive performance. To compare cognitive tests of patients and controls, Stroop test and TMT results were corrected for age and adjusted to a standard reference sample.

### Statistics

All statistical analyses were performed using IBM SPSS Statistics 22. A general linear mixed model (GLMM) was employed to determine significant main effects and interaction effects of independent variables on cortical thickness. The GLMM accounted for random intercepts of patients and hemispheres within patients, considering regions within hemispheres and patients as repeated measures. Non-significant interactions were hierarchically stepwise-backward eliminated from the model until only main effects and significant interactions remained. Two analyses were performed: first, including all 66 ROI, and second, including four vascular regions (ipsilateral and contralateral MCA region and non-MCA region). The following independent variables were included in the model: group (patient or control), hemisphere (left or right), presence of stenosis (yes or no), ROI and age. Group (patient or control) and stenosis (presence of stenosis yes or no) and hemisphere (left or right) were included as interaction terms to be able to identify potential specific regional effects of stenosis on individual hemispheres, thus enabling the separation of possible systematic effects of group from specific effects of unilateral stenosis on cortical thickness.

Results of cognitive tests were compared between groups using Student’s t-test or Mann-Whitney-U-test according to the distribution of the numbers. Correlation between test results and cortical thickness was investigated by Spearman correlation. Since this study was exploratory, no correction for multiple testing was performed. An alpha of 0.05 was considered significant.

## Results

Mean age of patients and control subjects was 67 (± 9.44) and 64 (± 8.36) years (*p* = 0.228). Of the 25 patients, 15 had a stenosis of the left carotid artery (cf. demographical table in the online Additional file 1: Table S1). Median degree of stenosis of the patient group was 80% (range 50–95%), all but one patient had stenosis ≥70%.

### General linear mixed model

In the GLMM including 66 anatomical ROI, ROI (*p* <  0.001) and age (*p* = 0.002) showed a significant effect on cortical thickness, while no significant effect was observed for hemisphere, group, and presence of stenosis (see Table 1). After hierarchical stepwise-backward elimination of non-significant interactions, no significant interaction remained. Estimated mean cortical thickness was numerically lower in the in the hemisphere with upstream carotid stenosis (with stenosis: 2.354; without: 2.363 mm, *p* = 0.180).

**Table 1:** Main effects of independent variables on cortical thickness in 66 Freesurfer ROIs; results of a linear mixed model analysis

Main effect	*P* value	Beta value [95% CI]
**ROI**	**< 0.001**	^**a**^ **[** ^**a**^ **]**
**Age (covariate)**	**0.002**	**−0.006 [(− 0.01)-(− 0.002)]**
Stenosis = no (no stenosis > stenosis)	0.180	0.009 [(− 0.004)-0.023]
Group = patients (patients < controls)	0.796	−0.009 [(− 0.08)-0.062]
Hemisphere = right (right < left)	0.937	−0.0004 [(− 0.01)-0.009]

^a^Beta values and 95% confidence intervals for each ROI can be found in the Additional file 1: Table S2

*ROI* region of interest, *CI* confidence interval

Results with significant *p* value were printed in bold

In the second model including MCA and non-MCA regions, region (*p* <  0.001), hemisphere (*p* = 0.003), and age (*p* = 0.009) showed a significant effect on cortical thickness, while stenosis did not (see Table 2). Estimated mean cortical thickness was higher in the MCA region as compared to the non-MCA region (2.344 mm vs. 2.021 mm, *p* <  0.001). There was a significant interaction between presence of stenosis and region (*p* = 0.042). Estimated mean cortical thickness in the MCA region in the hemisphere with upstream carotid stenosis was numerically lower than without stenosis (2.332 mm vs. 2.355 mm, *p* = 0.111).

**Table 2:** Main- and interaction effects of independent variables on cortical thickness in MCA and non-MCA region

Main effect	P value	Beta value [95% CI]
**Hemisphere = right (right < left)**	**0.003**	**−0.013 [(− 0.021)-(− 0.005)]**
**Age (covariate)**	**0.009**	**−0.005 [(− 0.009)-(− 0.001)]**
**Region = non-MCA (nonMCA < MCA)**	**< 0.001**	**−0.308 [(− 0.333)-(− 0.282)]**
Stenosis = no (stenosis< no stenosis)	0.34	0.023 [(− 0.005)-0.052]
**Stenosis = no & region = non-MCA (no stenosis nonMCA < stenosis non MCA, no stenosis MCA, stenosis MCA)**	**0.042**	**−0.031 [(− 0.06)-(− 0.001)]**
Group = patients (patients< controls)	0.637	−0.015 [(− 0.081)-0.05]

*MCA* middle cerebral artery, 95% CI = 95% confidence interval

Results with significant *p* value were printed in bold

### Cognitive tests

Stroop test C scores and TMT-B z scores were normally distributed for both groups. In the TMT B test, stenosis patients showed a trend towards lower Z-score values compared to controls (− 0.27 vs. 0.32, *p* = 0.054), indicating a longer time required and thus worse performance in the test. Regarding the Stroop test, patients had significant lower C-values (4.8 vs. 6.2, *p* = 0.010) indicating worse performance. Patients also achieved significantly worse test results for the CDT (1.52 vs. 1.04, *p* = 0.005) and DemTect (15.68 vs. 17.40, *p* = 0.009) (see Table 3). MMSE values were not normally distributed. Thus, a non-parametric test was used for group comparison. There was no significant difference between stenosis patients and controls (28.08 vs. 28.48, *p* = 0.375).

**Table 3:** Mean with standard deviation (in brackets) of C score of the Stroop Test, Z score of the TMT B and median with first and third quartile (in brackets) of MMSE, DemTect and CDT for both groups with *p* value for difference between the groups

	Patients	Controls	Group comparison (*p*-value)
Stroop Test, C score	4.780 (± 1.849)	6.2 (± 1.871)	**0.010** ^*^
TMT-B, Z score	−0.268 (± 1.112)	0.329 (± 1.023)	0.054^*^
MMSE	28 (27, 30)	29 (27, 30)	0.375^**^
DemTect	17 (14.5, 18)	18 (17, 18)	**0.009** ^**^
CDT	1 (1, 2)	1 (1, 1)	**0.005** ^**^

*: t test, **: non-parametrical test: Mann-Whitney-U test

*TMT-B* trail making test B, *MMSE* mini mental state examination, *DemTect* dementia detection test, *CDT* clock drawing test

In patients, no significant correlation between performance in cognitive and cortical thickness across the whole hemisphere, or any of the hemispheres (left or right, stenosis or no-stenosis) was observed (data not shown). In control subjects there also was no significant correlation between cognitive test results and cortical thickness except for a moderate correlation between DemTect result and cortical thickness of the left hemisphere (Spearman’s rank correlation coefficient = 0.403, *p* = 0.046).

## Discussion

In our study of patients with internal carotid artery stenosis without ischemic brain lesions, we did not observe significant alterations of cortical thickness in brain regions ipsilateral to the stenosis. While patients showed worse cognitive test performance than controls, there was no correlation of cognitive function and cortical thickness.

These results are in contrast to a recent study that reported a reduction of cortical thickness in the motor cortex of patients with carotid stenosis ≥80%, while no change of cortical thickness was detected in the visual cortex [12]. In the same patient cohort, a significant difference between cortical thicknesses on the side of stenosis versus non-stenosis side in M1 could be shown for the patients while this was not detectable in healthy controls [36]. A previous study also reported progressive brain atrophy measured by evaluation of brain volume associated with severe carotid stenosis in a longitudinal follow up of 4 years [37].

Worse performance of patients with carotid artery stenosis in cognitive tests in our study is in line with previous reports. Patients with either symptomatic or asymptomatic carotid stenosis showed worse results in cognitive tests than controls [19–22, 38], and lower MMSE scores were observed in patients with carotid stenosis ≥75% [14]. Findings from previous studies also support an association of grey matter volume and cognitive function independent from carotid stenosis. Smaller cortical gray matter volume was correlated with cognitive impairment, including among others tests for attention, memory and language and tests for executive performance [4, 39]. It was also hypothesized, that the association of brain volume and cognitive function might be mediated by cerebrovascular pathology including white matter lesions and brain infarcts^30^. We did not observe any meaningful correlation of cortical thickness with cognitive tests, although healthy controls achieved better results in the cognitive tests than stenosis patients. Other factors influencing cognitive function beyond cortical thickness may play a role leading to worse performance in patients, such as co-morbidities or vascular risk factors (cf., demographical data, Additional file 1: Table S1).

While cortical thickness provides important information on structural integrity of the brain, further techniques may be helpful to take into account other aspects of structural brain organization. In line with this, a study using diffusion tensor imaging observed reduced fractional anisotropy as a marker of white organization and diminished cognitive test results in patients with carotid stenosis [40]. The analysis of more complex parameters of brain structure including structural network organization may yield further insights into less pronounced structural sequelae of unilateral carotid stenosis and its interaction with cognitive function.

In our study, cortical thickness was associated with age, brain region, and hemisphere, which are well-described factors determining cortical thickness [5, 41]. We interpret the lack of obvious changes of cortical thickness in patents with carotid stenosis as an indicator of the robustness of the brain to chronic alterations of cerebral perfusion in the absence of ischemic brain lesions. Both, the network of collateral arteries and the mechanisms of neurovascular coupling may help render rain perfusion resilient and sufficient for maintaining the functional and structural metabolism within a wide range of changes to the cerebral blood flow. Only when focusing the analysis of cortical thickness changes in brain regions supplied by the middle cerebral artery, we observed an interaction of presence of stenosis and brain region, with slightly decreased cortical thickness in the MCA territory of brain hemispheres with upstream carotid stenosis. This interaction might indicate a small effect of unilateral carotid stenosis on cortical thickness beyond the effects age, brain region, and hemisphere. Our sample, however, may have been too small, to detect this small effect in the statistical model applied.

## Conclusion

To summarize, in patients with carotid stenosis without ischemic brain lesions we observed neither significantly reduced cortical thickness nor an association of cortical thickness with cognitive function, although patients performed worse than healthy controls in cognitive tests. These data do not support the hypothesized association of cortical thinning and cognitive impairment in carotid stenosis. In contrast, our results demonstrate the robustness of brain structure to chronic changes of cerebral perfusion in the absence of focal stroke lesions. Future studies should take into account a wider range of clinical and imaging parameters when studying the effects of alterations of cerebral perfusion on brain structure and cognitive function.

## Additional file

Additional file 1: **Table S1**. Demographic data stratified by group. **Table S2**. Beta values and 95% confidence interval for each region of interest. (DOCX 17 kb)

## Data Availability

The datasets used and/or analysed during the current study are available from the corresponding author on reasonable request.

## Abbreviations

CDT: Clock drawing test
CERAD: Consortium to establish a registry for Alzheimer’s disease
CI: Confidence interval
DemTect: Dementia detection test
FOV: Field of view
FWT: Farb-Wort-test
GLMM: General linear mixed model
IPR: In-plane resolution
MCA: Middle cerebral artery
MMSE: Mini mental state examination
MPRAGE: Magnetization-prepared rapid acquisition gradient-echo
n/a: Not applicable
NASCET: North American symptomatic carotid Endarterectomy trial
ROI: Region of interest
SD: Standard deviation
ST: Slice thickness
TE: Echo time
TMT: Trail making test
TR: Repetition time

## References

[1] Flaherty ML, Kissela B, Khoury JC, Alwell K, Moomaw CJ, Woo D (2013). Carotid artery stenosis as a cause of stroke. Neuroepidemiology..

[2] Yuan Guiyi, Zhou Shuxian, Wu Wei, Zhang Yuling, Lei Juan, Huang Boshui (2018). Carotid Artery Stenting Versus Carotid Endarterectomy for Treatment of Asymptomatic Carotid Artery Stenosis. International Heart Journal.

[3] Moresoli P, Habib B, Reynier P, Secrest MH, Eisenberg MJ, Filion KB (2017). Carotid stenting versus Endarterectomy for asymptomatic carotid artery stenosis: a systematic review and meta-analysis. Stroke..

[4] Kim HJ, Ye BS, Yoon CW, Noh Y, Kim GH, Cho H (2014). Cortical thickness and hippocampal shape in pure vascular mild cognitive impairment and dementia of subcortical type. Eur J Neurol.

[5] Lemaitre H, Goldman AL, Sambataro F, Verchinski BA, Meyer-Lindenberg A, Weinberger DR (2012). Normal age-related brain morphometric changes: nonuniformity across cortical thickness, surface area and gray matter volume?. Neurobiol Aging.

[6] Li Q, Pardoe H, Lichter R, Werden E, Raffelt A, Cumming T (2015). Cortical thickness estimation in longitudinal stroke studies: a comparison of 3 measurement methods. NeuroImage Clin..

[7] Cardenas VA, Reed B, Chao LL, Chui H, Sanossian N, DeCarli CC (2012). Associations among vascular risk factors, carotid atherosclerosis, and cortical volume and thickness in older adults. Stroke..

[8] Jones PW, Borich MR, Vavsour I, Mackay A, Boyd LA (2016). Cortical thickness and metabolite concentration in chronic stroke and the relationship with motor function. Restor Neurol Neurosci.

[9] Zhang J, Meng L, Qin W, Liu N, Shi FD, Yu C (2014). Structural damage and functional reorganization in Ipsilesional M1 in well-recovered patients with subcortical stroke. Stroke..

[10] Cheng B, Schulz R, Bönstrup M, Hummel FC, Sedlacik J, Fiehler J (2015). Structural plasticity of remote cortical brain regions is determined by connectivity to the primary lesion in subcortical stroke. J Cereb Blood Flow Metab.

[11] Fierstra J, Poublanc J, Han JS, Silver F, Tymianski M, Crawley AP (2010). Steal physiology is spatially associated with cortical thinning. J Neurol Neurosurg Psychiatry.

[12] Marshall Randolph S., Asllani Iris, Pavol Marykay A., Cheung Ying-Kuen, Lazar Ronald M. (2017). Altered cerebral hemodyamics and cortical thinning in asymptomatic carotid artery stenosis. PLOS ONE.

[13] Avelar WM, D’Abreu A, Coan AC, Lima FO, Guimarães R, Yassuda CL (2015). Asymptomatic carotid stenosis is associated with gray and white matter damage. Int J Stroke.

[14] Johnston SC, O’Meara ES, Manolio TA, Lefkowitz D, O’Leary DH, Goldstein S (2004). Cognitive impairment and decline are associated with carotid artery disease in patients without clinically evident cerebrovascular disease. Ann Intern Med.

[15] Balucani C, Viticchi G, Falsetti L (2012). Cerebral hemodynamics and cognitive performance in bilateral asymptomatic carotid stenosis.

[16] Mathiesen EB, Waterloo K, Joakimsen O, Bakke SJ, Jacobsen EA, Bønaa KH (2004). Reduced neuropsychological test performance in asymptomatic carotid stenosis: the Tromsø study. Neurology..

[17] Mendiz OA, Sposato LA, Fabbro N, Lev GA, Calle A, Valdivieso LR (2012). Improvement in executive function after unilateral carotid artery stenting for severe asymptomatic stenosis. J Neurosurg.

[18] Landgraff NC, Whitney SL, Rubinstein EN, Yonas H (2010). Cognitive and physical performance in patients with asymptomatic carotid artery disease. J Neurol.

[19] Wang T, Xiao F, Wu G, Fang J, Sun Z, Feng H (2017). Impairments in brain perfusion, metabolites, functional connectivity, and cognition in severe asymptomatic carotid stenosis patients: an integrated MRI study. Neural Plast.

[20] Norling AM, Marshall RS, Pavol MA, Howard G, Howard V, Liebeskind D, et al. Is Hemispheric Hypoperfusion a Treatable Cause of Cognitive Impairment? Curr Cardiol Rep [Internet]. Current Cardiology Reports; 2019;21(1):4. Available from: http://www.ncbi.nlm.nih.gov/pubmed/30661122. 10.1007/s11886-019-1089-9 PMC8985657 30661122

[21] Alhusaini S, Karama S, Nguyen TV, Thiel A, Bernhardt BC, Cox SR (2018). Association between carotid atheroma and cerebral cortex structure at age 73 years. Ann Neurol.

[22] Lal BK, Dux MC, Sikdar S, Goldstein C, Khan AA, Yokemick J (2017). Asymptomatic carotid stenosis is associated with cognitive impairment. J Vasc Surg.

[23] Rimmele DL, Larena-Avellaneda A, Alegiani AC, Rosenkranz M, Schmidt NO, Regelsberger J (2017). Real-world experience of treatment decision-making in carotid stenosis in a neurovascular board. Neurology..

[24] Cohen S, Tyrrell D, Smith A (1991). Beneficial effect of carotid Endarterectomy in symptomatic patients with high-grade carotid stenosis. N Engl J Med.

[25] Hathout GM, Fink JR, El-Saden SM, Grant EG (2005). Sonographic NASCET index: a new doppler parameter for assessment of internal carotid artery stenosis. AJNR Am J Neuroradiol.

[26] Fischl B (2012). FreeSurfer. Neuroimage2.

[27] Dale AM, Fischl B, Sereno MI (1999). Cortical surface-based analysis. I. Segmentation and surface reconstruction. Neuroimage..

[28] Fischl B, Dale AM (2000). Measuring the thickness of the human cerebral cortex from magnetic resonance images. Proc Natl Acad Sci.

[29] Wu J, Ngo GH, Greve D, Li J, He T, Fischl B, et al. Accurate nonlinear mapping between MNI volumetric and FreeSurfer surface coordinate systems. Hum Brain Mapp. 2018;(April:1–16. 10.1002/hbm.24213 PMC6239990 29770530

[30] Fischl B, Sereno MI, Tootell RBH (1999). Dale a M. high-resolution inter-subject averaging and a surface-based coordinate system. Hum Brain Mapp.

[31] Tatu LMD, Moulin TMD, Bogousslavsky JMD, Duvernoy HMD (1998). Arterial territories of the human brain: cerebral hemispheres. Neurology..

[32] Mutsaerts HJMM, Richard E, Heijtel DFR, Van Osch MJP, Majoie CBLM, Nederveen AJ (2014). Gray matter contamination in arterial spin labeling white matter perfusion measurements in patients with dementia. NeuroImage Clin.

[33] Cheng B, Golsari A, Fiehler J, Rosenkranz M, Gerloff C, Thomalla G (2011). Dynamics of regional distribution of ischemic lesions in middle cerebral artery trunk occlusion relates to collateral circulation. J Cereb Blood Flow Metab.

[34] Ashendorf L, Jefferson A, Oconnor M, Chaisson C, Green R, Stern R (2008). Trail making test errors in normal aging, mild cognitive impairment, and dementia. Arch Clin Neuropsychol.

[35] Oswald WD, Fleischmann UM (1997). Das Nürnberger-Alters-Inventar.

[36] Asllani I, Slattery P, Fafard A, Pavol M, Lazar RM, Marshall RS (2016). Measurement of cortical thickness asymmetry in carotid occlusive disease. NeuroImage Clin..

[37] Muller M, Van Der Graaf Y, Algra A, Hendrikse J, Mali WP, Geerlings MI (2011). Carotid atherosclerosis and progression of brain atrophy: the SMART-MR study. Ann Neurol.

[38] Jackson DC, Sandoval-Garcia C, Rocque BG, Wilbrand SM, Mitchell CC, Hermann BP (2016). Cognitive deficits in symptomatic and asymptomatic carotid Endarterectomy surgical candidates. Arch Clin Neuropsychol.

[39] Muller M, Appelman APA, van der Graaf Y, Vincken KL, Mali WPTM, Geerlings MI (2011). Brain atrophy and cognition: interaction with cerebrovascular pathology?. Neurobiol Aging.

[40] Cheng H-L, Lin C-J, Soong B-W, Wang P-N, Chang F-C, Wu Y-T (2012). Impairments in cognitive function and brain connectivity in severe asymptomatic carotid stenosis. Stroke..

[41] Luders E, Narr KL, Thompson PM, Rex DE, Jancke L, Toga AW (2006). Hemispheric asymmetries in cortical thickness. Cereb Cortex.

[42] CERAD-Plus Online (https://www.memoryclinic.ch/de/main-navigation/cerad-plus/), of the Memory Clinic (Universitäre Altersmedizin, Felix Platter-Spital, Burgfelderstrasse 101, Postfach, 4002 Basel, Tel. +41 61 326 47 60, memoryclinic@fps-basel.ch).

