# Supplementary material for: Cortical thickness and cognitive performance in asymptomatic unilateral carotid artery stenosis
Source: BMC Cardiovasc Disord. 2019 Jun 25;19:154. doi: 10.1186/s12872-019-1127-y (PMC6593546; doi:10.1186/s12872-019-1127-y)
Supplement: Supplementary file 1 — Table S1. Demographic data stratified by group. Table S2. Beta values and 95% confidence interval for each region of interest. (DOCX 17 kb) [file 12872_2019_1127_MOESM1_ESM.docx]

Additional file 1

*Table S1: Demographic data stratified by group*

| Group | Patients n = 25 | Healthy controls n = 25 |
| --- | --- | --- |
| Age, mean ± SD | 66.8 ± 9.4 | 63.7 ± 8.4 |
| Sex = female, n (%) | 7 (28 %) | 9 (36 %) |
| Arterial hypertension, n (%) | 20/25 (80 %) | 4/14 (28.6 %) |
| Diabetes mellitus | 5/25 (20 %) | 2/14 (14.3 %) |
| Hyperlipidemia | 12/25 (48 %) | 4/14 (28.6 %) |
| Nicotine abuse | 9/24 (37.5 %) | 2/15 (13.3 %) |
| Years of education, mean ± SD | 15 ± 4.2 (3 n/a) | 16.4 ± 3.4 (1 n/a) |

SD = standard deviation

n/a = not applicable, no

*Table S2: Beta values and 95 % confidence interval for each region of interest*

|  | **Coefficient** | **95 % CI** | **95 % CI** |
| --- | --- | --- | --- |
| **ROI** |  | **lower boundary** | **upper boundary** |
| [ROI=bankssts_thickness] | 0.24264 | 0.185845681 | 0.299434319 |
| [ROI=caudalanteriorcingulate_thickness] | 0.44334 | 0.368291799 | 0.518388201 |
| [ROI=caudalmiddlefrontal_thickness] | 0.1867 | 0.133509348 | 0.239890652 |
| [ROI=cuneus_thickness] | -0.50833 | -0.562609121 | -0.454050879 |
| [ROI=frontalpole_thickness] | 0.47258 | 0.40360046 | 0.54155954 |
| [ROI=fusiform_thickness] | 0.48028 | 0.425948206 | 0.534611794 |
| [ROI=inferiorparietal_thickness] | 0.11977 | 0.069147049 | 0.170392951 |
| [ROI=inferiortemporal_thickness] | 0.65165 | 0.597773824 | 0.705526176 |
| [ROI=insula_thickness] | 0.74094 | 0.685909448 | 0.795970552 |
| [ROI=isthmuscingulate_thickness] | 0.14212 | 0.079514398 | 0.204725602 |
| [ROI=lateraloccipital_thickness] | -0.12153 | -0.173168543 | -0.069891457 |
| [ROI=lateralorbitofrontal_thickness] | 0.34559 | 0.291503502 | 0.399676498 |
| [ROI=lingual_thickness] | -0.28891 | -0.341296526 | -0.236523474 |
| [ROI=medialorbitofrontal_thickness] | 0.24755 | 0.18914213 | 0.30595787 |
| [ROI=middletemporal_thickness] | 0.64444 | 0.590905948 | 0.697974052 |
| [ROI=paracentral_thickness] | 0.01363 | -0.040674528 | 0.067934528 |
| [ROI=parahippocampal_thickness] | 0.55059 | 0.47942413 | 0.62175587 |
| [ROI=parsopercularis_thickness] | 0.21456 | 0.160840036 | 0.268279964 |
| [ROI=parsorbitalis_thickness] | 0.3307 | 0.270881026 | 0.390518974 |
| [ROI=parstriangularis_thickness] | 0.09797 | 0.043707302 | 0.152232698 |
| [ROI=pericalcarine_thickness] | -0.75991 | -0.814191445 | -0.705628555 |
| [ROI=postcentral_thickness] | -0.28266 | -0.333705599 | -0.231614401 |
| [ROI=posteriorcingulate_thickness] | 0.20382 | 0.150699685 | 0.256940315 |
| [ROI=precentral_thickness] | 0.14122 | 0.087121607 | 0.195318393 |
| [ROI=precuneus_thickness] | -0.00193 | -0.054266768 | 0.050406768 |
| [ROI=rostralanteriorcingulate_thickness] | 0.63017 | 0.561436056 | 0.698903944 |
| [ROI=rostralmiddlefrontal_thickness] | 0.04424 | -0.008119856 | 0.096599856 |
| [ROI=superiorfrontal_thickness] | 0.42133 | 0.369268227 | 0.473391773 |
| [ROI=superiorparietal_thickness] | -0.16505 | -0.216242736 | -0.113857264 |
| [ROI=superiortemporal_thickness] | 0.51473 | 0.460743579 | 0.568716421 |
| [ROI=supramarginal_thickness] | 0.18228 | 0.130145445 | 0.234414555 |
| [ROI=temporalpole_thickness] | 1.42058 | 1.345580769 | 1.495579231 |
| [ROI=transversetemporal_thickness] | 0* |  |  |

* Value for Reference

95 % CI= 95 % Confidence interval

ROI = region of interest
